# Postoperative Outcomes of Endoscopic versus Microscopic Myringoplasty in Patients with Chronic Otitis Media—A Systematic Review

**DOI:** 10.3390/medicina59061074

**Published:** 2023-06-02

**Authors:** Iemima Stefan, Cristian Dragos Stefanescu, Ana Maria Vlad, Viorel Zainea, Răzvan Hainarosie

**Affiliations:** 1 Medical Center of Special Telecommunications Service, 060044 Bucharest, Romania; 2 ENT Department, Faculty of Medicine, “Carol Davila” University of Medicine and Pharmacy, 030167 Bucharest, Romania; 3 “Prof. Dr. Dorin Hociota” Institute of Phonoaudiology and Functional ENT Surgery, 21st Mihail Cioranu Street, 061344 Bucharest, Romania

**Keywords:** endoscopic, microscopic, myringoplasty, chronic otitis media, review

## Abstract

Endoscopes are increasingly being used in middle ear surgery as an adjunct to or replacement for the operative microscope. The superior visualization of hidden areas and a minimally invasive transcanal approach to the pathology are some of the endoscope’s advantages. The aim of this review is to compare the surgical outcomes of a totally endoscopic transcanal approach with a conventional microscopic approach for type 1 tympanoplasty in patients with chronic otitis media (COM) in order to establish if endoscopic myringoplasty (EM) could be a better alternative to microscopic myringoplasty (MM). A literature review was performed using the Preferred Reporting Items for Systematic Reviews and Meta-Analysis recommendations. The selected articles were identified by searching PubMed Central, PubMed, MEDLINE and Embase databases for the relevant publications. Only studies where the same surgeon in the department performed both endoscopic and microscopic myringoplasty have been included in the review. The results suggest that with an endoscopic approach, minimally invasive myringoplasty can be achieved with a similar graft success rate and postoperative air–bone gap (ABG) improvement, a shorter operative time and less postoperative complications compared to a microscopic approach.

## 1. Introduction

Chronic otitis media (COM) is a complex multifactorial inflammatory and infectious condition that is mainly characterized by middle ear mucosal inflammation with permanent tympanic membrane perforation and, in some cases, fixation or interruption of the ossicular chain.

The main objectives in treating chronic otitis media are to repair the tympanic membrane perforation, eradicate chronic infection and, if necessary, restore the integrity and mobility of the ossicular chain.

Microscopic myringoplasty (MM) has been the standard surgery for repairing perforated tympanic membranes since the 1950s, but since the late 1990s, endoscopic myringoplasty (EM) has been increasingly practiced.

The *MM approach* offers binocular vision along with an excellent stereoscopic surgical view and leaves both surgeons’ hands free, but it is limited by the straight-line vision that makes the visualization of the middle ear through the ear canal relatively difficult [1]. Therefore, conventional MM is originally performed using a postauricular incision, with or without drilling of the bony ear canal, in order to obtain adequate visualization and illumination [2]. A postauricular incision may produce surgical scarring, temporary loss of cutaneous sensation [3] and malposition of the ear.

The *EM approach* has several advantages when compared to the conventional postauricular MM: it avoids unnecessary incisions and soft tissue dissections, ensures easy access to hidden areas, eliminates the potential need for canalplasty, provides a shorter operative time and has lower complication rates [4,5,6,7].

In the last years, there have been several comparative studies published regarding the efficacy of the two operative approaches [2,5,8,9,10,11,12], but few systematic reviews regarding the comparison between endoscopic and microscopic tympanoplasty [13].

Therefore, we decided to perform a systematic review to compare the postoperative outcomes of the totally endoscopic transcanal approach with a conventional microscopic approach for myringoplasty in patients with COM in order to analyze if the endoscopic approach can provide at least the same results as the microscopic approach or even better outcomes in terms of graft success rates, hearing improvement, operative time and postoperative complications, and to establish if EM could be a better alternative to MM. 

## 2. Materials and Methods

This systematic review was conducted in accordance with the Preferred Reporting Items for Systematic Reviews and Meta-Analyses (PRISMA) guidelines [14]. No ethical approval was required as previously published studies were analyzed. The PICO framework was used to develop the specific research question:

1. Population: patients with chronic otitis media
2. Intervention: myringoplasty
3. Comparison: an endoscopic versus a microscopic approach
4. Outcome: graft success rate, air–bone gap (ABG) improvement, operative time, postoperative complications

### 2.1. Search Strategy

Two of the authors independently searched the PubMed Central, PubMed, MEDLINE and Embase databases for relevant publications on 8 March 2023. We searched for all available studies reporting comparisons between endoscopic type 1 tympanoplasty or myringoplasty and microscopic type 1 tympanoplasty or myringoplasty in patients with chronic otitis media. The following keywords were used for searching through the PubMed Central database: “((((endoscopic) AND microscopic)) AND (((myringoplasty) OR tympanoplasty) OR type 1 tympanoplasty)) AND ((chronic otitis media) OR COM)”.

### 2.2. Selection of Studies

**Figure 1:**
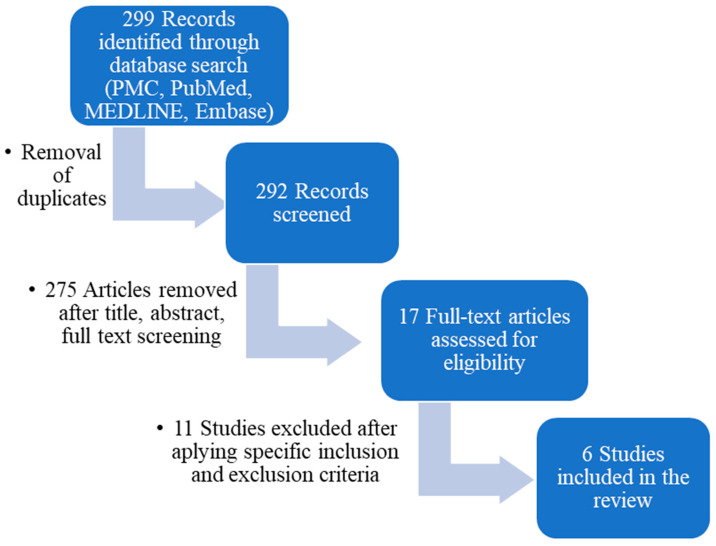
Preferred Reporting Items for Systematic Reviews and Meta-Analyses (PRISMA) flow diagram outlining the study design.

Only full-text English studies were selected, with no restriction regarding the date of publication. All duplicates were manually removed before the study titles and abstracts were screened. Lastly, the remaining studies had their full texts reviewed. The full texts of eligible articles were subsequently evaluated based on the inclusion and exclusion criteria (Figure 1).

The inclusion criteria consisted of: (1) patients with chronic suppurative otitis media with inactive disease and an intact ossicular chain; (2) studies comparing endoscopic with microscopic myringoplasty; (3) studies where the same surgeon carried out both endoscopic and microscopic myringoplasty; (4) studies providing outcome measures such as graft success rate, audiometric outcomes, or duration of surgery in both the endoscopic and microscopic groups.

The following exclusion criteria were applied: (1) studies including patients with cholesteatoma, ossicular chain disorders, adhesive/atelectatic otitis media, active/granulative COM; (2) studies performing simultaneous otologic procedures in addition to myringoplasty (e.g., ossicular chain reconstruction, mastoidectomy); (3) studies with non-available English full-text, duplicate publications, publications where original articles were inaccessible (e.g., only abstracts were available) and/or incomplete data were provided; (4) animal studies, in vitro studies; (5) review articles, case reports; (6) studies with only one approach assessed (only EM or only MM) or a mixture of the two (endoscope-assisted microscopic myringoplasty, microscopic-assisted endoscopic myringoplasty); and (7) subjects unrelated to the researched topic.

### 2.3. Data Extraction and Management

Two authors reviewed all the relevant studies and independently extracted the data; any discrepancies were resolved by consensus between the two authors.

For each selected article, the following information was noted in a template built for this study: the author, year of publication, period of the study, type of study, number of patients in total and in each comparative group, mean age of each comparative group, mean follow-up period in each group (Table 1), location and size of perforation, graft material, and graft technique in each comparative group (Table 2), graft success rate for each group, mean pre- and postoperative air–bone gap in each group, mean operative time for each approach (Table 3) and postoperative complications in each group. 

## 3. Results

### 3.1. Results of the Literature Search

There were 299 articles identified in the databases. After the removal of duplicates, 292 studies remained. These studies were screened via the title, abstract and full-text, when needed, for relevance, leaving 17 studies for full-text review. Applying all the inclusion and exclusion criteria, there were another 11 studies excluded at this stage, leaving 6 eligible studies to be included in the present article [15,16,17,18,19,20]. 

The PRISMA flow diagram used to describe the flow of information throughout various phases of the systematic review is displayed in Figure 1. 

### 3.2. Characteristics of the Included Studies

From the selected articles, five were retrospective non-randomized studies and one was a prospective randomized controlled trial. 

The enrolled studies were conducted between 2008 and 2018. Four of the studies comprised over 100 patients each, and two of them had fewer than 100.

The total number of patients in the present review is 859 and the total number of ears analyzed is 887. Of these, 57.38% (n = 509) underwent EM and 42.62% (n = 378) MM. 

*The age* of patients ranged from 15 to 77 years, with no significant difference in the mean age between the two comparative groups in each study.

*Sex distribution.* Overall, 47.26% (n = 406) of patients were male and 52.74% (n = 453) were female. In the EM groups, 50.5% (n = 250) of patients were male and 49.5% (n = 245) were female, whereas in the MM groups 42.86% (n = 156) of patients were male and 57.14% (n = 208) were female. 

*Perforation characteristics* were assessed in four out of six studies with respect to its anatomic localization and size. 

Regarding their anatomic localization, tympanic membrane perforations were grouped as central, marginal, anterior and posterior. In two studies, central perforations were more often described [16,18], whereas in one study, anterior perforations were more frequent, with no significant differences between the EM and MM groups [15]. With respect to the perforation size, they were classified as small, medium/moderate, large, subtotal or total, and the distribution of perforation size between the EM and MM groups was assessed. In one study [15], perforations of the tympanic membrane were classified as small (perforation of the tympanic membrane less than 25%), medium (between 25% and 75%) or large (more than 75%). The distribution of perforation size was similar between the EM and MM groups, with no statistically significant differences: 53.8% small, 29.5% medium and 16.7% large perforations in the EM group, and 54.9% small, 30.8% medium and 14.1% large perforations in the MM group. In the second study [16], the size of the perforation was assessed in the same way: small (<25%), moderate (25% to 75%) or large (>75%) of the surface of the tympanic membrane, although the distribution between the two groups was not described, but only general distribution, 18.5% small, 46.2% moderate and 35.4% large perforations. In the third study [17], all ears included in the study had subtotal perforation. Another study [18] included only large-sized perforations: 50–75% (large) or >75% (subtotal) of the pars tensa, with even distribution between the EM and MM groups: 72.7% large perforations and 27.3% subtotal perforations in the EM group and 76.2% large perforations and 23.8% subtotal perforations in the MM group. 

*Surgical approach*. While for all subjects in the EM group the transcanal approach was utilized, those with MM were approached using the retroauricular technique. The surgeries in each study (both endoscopic and microscopic myringoplasties) were performed by the same surgeon.

**Figure 2:**
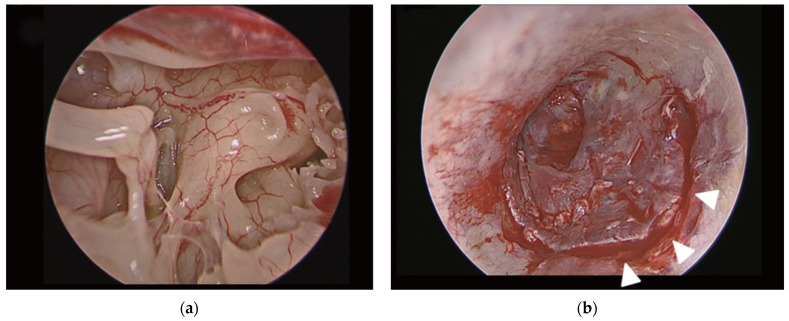
(**a**) Endoscopic view of middle ear cavity during endoscopic myringoplasty; (**b**) view of transcanal incision (white arrow heads) in endoscopic myringoplasty. Final aspect of endoscopic myringoplasty. Adapted with the permission from Ref. [2]. Copyright © 2017 by Korean Society of Otorhinolaryngology-Head and Neck Surgery.

EM surgical technique (Figure 2). Perforation edges were freshened. Transcanal incisions were made. The tympanomeatal flap was elevated. The fibrous annulus was separated from the tympanic sulcus with the preservation of the chorda tympani nerve, and the middle ear space was reached. The mobility and integrity of the ossicular chain were checked by gentle palpation of the ossicles. The graft was positioned using an underlay technique. The tympanomeatal flap was placed in its original position and tightly supported with Gelfoam.

**Figure 3:**
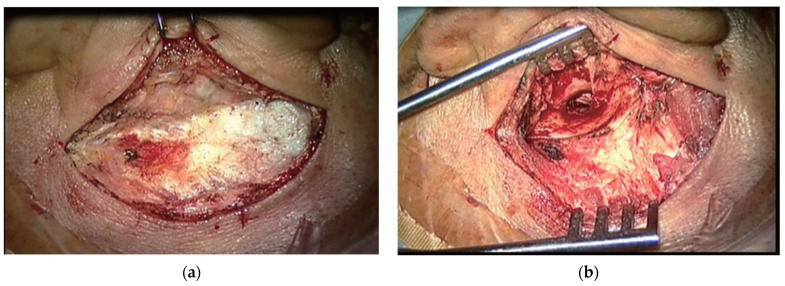
(**a**) Postauricular incision for microscopic postauricular myringoplasty. (**b**) Microscopic view of operation field of myringoplasty. Adapted with the permission from Ref. [2]. Copyright © 2017 by Korean Society of Otorhinolaryngology-Head and Neck Surgery.

MM surgical technique (Figure 3). The perforation edges were freshened. A postauricular Wilde’s incision was performed. The tympanomeatal flap was elevated under the guidance of the surgical microscope. A canalplasty was practiced, when needed, by drilling the bony ear canal. The mobility and the integrity of the ossicular chain were checked. The graft was positioned with the underlay technique. The tympanomeatal flap was placed in its original position and supported with Gelfoam. The postaural wound was sutured and a compressive dressing was applied over it.

*The graft material* consisted of temporalis fascia, postconchal or tragal perichondrium, full-thickness tragal chondroperichondial graft, or a 0.5 mm-thickness conchal chondroperichondrial graft (Table 2). 

*The graft technique.* An underlay graft technique was the preferred method in five studies, while one study used the over–underlay technique [18].

The *follow-up period* extended between 6 and 12 months.

*Outcomes assessed.* All studies included data regarding the following outcomes: graft success rate, hearing outcome in terms of pre- and postoperative air–bone gap and the mean operative time, in both groups. All the studies, except one, provided information about the postoperative complications [15,16,17,18,20]. 

### 3.3. Outcomes Analysis (Table 3)

#### 3.3.1. Graft Success Rate 

The graft success rate ranged between 86.30% [18] and 98% [19] in the EM group and between 85.70% [18] and 98% [19] in the MM group. There were no statistically significant differences found between the graft success rates in the EM group and the MM group in each study (Table 2).

#### 3.3.2. Pre-Op and Post-Op ABG

In all studies, Pure Tone Audiometry (PTA) was performed pre- and postoperatively at frequencies of 500, 1000, 2000 and 4000 Hz to determine the Air Conduction (AC) thresholds, Bone Conduction (BC) thresholds and air–bone gap (ABG) values. 

Pre-op ABG values ranged from 20.7 dB [18] to 34.16 dB [20] in the EM group and from 17.6 dB [18] to 35.54 dB [20] in the MM group. On the other hand, post-op ABG values ranged from 7.72 dB [17] to 18 dB [20] in the EM group and from 7.9 dB [15] to 16 dB [20] in the MM group. One study found the difference between hearing improvement in the EM group and the MM group being statistically significant at 1 month follow-up in favor of the EM group (*p* = 0.063 [15]), but not at 12 months, and one study found the difference between ABG improvement in the EM group and the MM group being statistically significant at 12 months follow-up in favor of the MM group (*p* = 0.0001 [20]).

#### 3.3.3. Mean Operative Time

All studies reported operation time data. The EM group had a shorter mean operative time than the MM group; this finding was consistent in all studies and statistically significant in five of six studies (*p* < 0.05 [15]; *p* < 0.001 [16]; *p* < 0.0001 [17]; *p* = 0.006 [18]; *p* < 0.0001 [19]). The mean operative time in the EM group varied between 34.9 [15] and 79.8 min [18] and between 52.7 [15] and 161 min [16] in the MM group. 

#### 3.3.4. Postoperative Complications

Four of the six studies [15,16,17,20] investigated postoperative complications that appeared in each group. The reported postoperative complications in the MM groups included: numbness around the ear [15,17], tinnitus [17], wet ear/granulation tissue [16,17,20], abnormal taste/dysgeusia [15,17], postauricular hematoma, wound infection, otitis externa, asymmetry of the auricle and wound dehiscence [15]. For the EM groups, the reported postoperative complications included: tinnitus [17], wet ear/granulation tissue [20], abnormal taste/dysgeusia [15,17] and otitis externa [15].

Postoperative pain was assessed in four studies [16,17,18,20]; one of them used the WILDA’s pain assessment guide [20], two studies used the visual analog scaling (VAS) method (0 for no pain and 10 for the worst pain imaginable) [16,18] and one study only mentioned the percentage of patients that experienced ear pain postoperatively, irrespective of its intensity [17]. In the first case, the pain score was found to be 5 in the MM group as compared to 4 in the EM group [20]. In the studies using the VAS method, the pain scale scores did not differ significantly between the groups in one study [18], while postoperative pain was found to be significantly lower in patients who underwent endoscopic surgery (*p* < 0.001) in the other one [16].

### 3.4. Subgroup Analysis

#### 3.4.1. Graft Material

Graft material varied between groups and between studies, consisting of either temporalis fascia, postconchal or tragal perichondrium, full-thickness tragal chondroperichondial graft or a 0.5 mm-thickness conchal chondroperichondrial graft. Temporalis muscle fascia tended to be used more frequently in the MM group [17,18,19,20], as the approach was postaural and the graft place was close to the incision site. Tragal/conchal perichondrium as well as tragal/conchal chondroperichondial grafts were used in the EM group [15,16,17,18], the last being also used in the MM group in two studies [15,16].

##### Graft Success Rate

Groups with temporalis fascia grafts had a graft success rate varying from 85.70% [18] to 98% [20], with both the lowest and highest graft success rate values belonging to the MM group. 

The graft success rate in groups with tragal chondroperichondrial grafts was between 92.90% in the MM group and 94.80% in the EM group [15], while for conchal chondroperichondrial grafts, success rates varied between 96.40% in the MM group and 97.30% in the EM group [16]. 

Postconchal perichondrium had a graft success rate of 86.30% [18] and tragal perichondrium of 98% [19], both being surgically placed using an endoscopic approach.

##### Post-Op AGB 

For the temporalis muscle fascia graft, a post-op ABG of 8.34 to 18 dB was noted, with smaller post-op ABGs for MM groups [17,18,19,20]. 

For tragal chondroperichondrial grafts, the smallest post-op ABG was in an EM group (7.72 dB) [17], but similar to the MM group from another study (7.9 dB) [15]. 

Conchal chondroperichondrial grafts had a comparable mean post-op ABG between the EM and MM groups from the same study: 15.7 dB and 14.4 dB [16]. 

The smallest mean post-op ABG in the present review was achieved using a tragal chondroperichondrial graft (7.72 dB [17]) and the highest using temporalis fascia (18 dB [20]). Another study revealed very good results regarding post-op ABG using temporalis fascia grafts, of 8.34 dB [17]. Further studies are needed to compare the outcomes of myringoplasty in correlation with the graft material used.

##### Mean Operative Time

The shortest operative times were noted in both the EM and MM groups that used tragal chondroperichondrial grafts, 34.9 and 52.7 min [15], while the longest mean operative times were needed for myringoplasties that used temporalis fascia as graft material: 120 min, 99.9 min, 81.22 min and 75.5 min [17,18,19,20]; but also for 0.5 mm-thickness conchal chondroperichondrial graft, having the longest mean operative time in MM groups: 161 min [16] and almost the longest mean operative time in EM groups: 76.7 min [16].

Nonhomogenous data from the studies on postoperative complications did not allow us to conduct a subgroup analysis for this specific outcome.

#### 3.4.2. Graft Technique 

All studies included in the review used the underlay graft technique, except one study that used the over–underlay technique [18]. Looking at this specific study’s outcomes in comparison with the other five studies included in the review, the following observations can be made: it has the lowest graft success rates in both the EM and MM groups, the highest mean operative time in EM groups, but satisfactory post-op ABGs in both groups. However, the results cannot be generalized because this study consisted of a small number of patients, collected over a long period of time, including patients from the beginning of the endoscopic era. Further studies are needed to elucidate which graft technique has better outcomes. 

## 4. Discussion

The present review comprised a relatively large number of patients and it revealed a comparable graft success rate between endoscopic and microscopic approaches. The graft success rate via the EM ranged from 86.30% to 98%, which is consistent with that of the MM. There were no statistically significant differences found between graft success rates in the EM group and the MM group in each study.

Age is a factor previously associated with the graft success rate and it might influence the results [21]. In the present review, studies that had similar age distribution between the two groups (EM and MM) were included.

Previous studies suggested that the comparable graft success rate is more likely associated with the grafting technique than surgical approach [22]. In this review, all authors, except one, used the underlay graft technique. Additional studies comparing the different grafting techniques might clarify this aspect.

The graft material could also be considered a variable that might influence postoperative outcomes. Conceptually, one might anticipate significant conductive hearing loss in cartilage myringoplasty, especially in the lower tones, with a tympanic membrane that is rigid and thick. However, in several studies and meta-analyses, the subgroup of full-thickness cartilage grafts revealed a slight, but significantly superior, hearing outcome than the temporalis fascia graft group [23]. Moreover, Gerber et al. demonstrated that cartilage does not impede sound transmission [24]. Four meta-analyses [25,26,27,28] and one systemic review [29] showed no difference regarding audiometric results between the cartilage and temporalis fascia grafts. More research is needed in this direction as well. The hearing improvements of EM and MM were comparable, which supported previous studies, suggesting that an endoscopic approach can be a good alternative to microscopic technique for patients with COM that require myringoplasty. In our review, the EM was superior to the MM in terms of operation time and postoperative complications. The mean operative time in EM groups was significantly shorter than that in MM groups. Suturing the postauricular incision in the MM group might extend the operation time. Moreover, postoperative complications seemed to be more likely to appear in the MM group, and they were mostly related to the postaural incision. On the other hand, sometimes, bleeding may be an inconvenience and a time-consuming factor during the endoscopic approach, that could even lead, in some cases, to a conversion to the microscopic approach. Yet, we found the shortest average operative time in EM groups, which is consistent with relevant studies in the literature, and no study included in our review reported incomplete endoscopic surgery with a microscopic conversion due to bleeding. The management of bleeding in endoscopic ear surgery is feasible through widely available hemostatic agents such as the injection of diluted epinephrine, cottonoids soaked with epinephrine (1:1000), mono- or bipolar cautery, washing with hydrogen peroxide, and self-suctioning instruments, and even the highest bleeding scores could be managed in an exclusively endoscopic technique in a study conducted by Anschuetz L. et al. [30]

Looking beyond myringoplasty, the place of the endoscopic approach in middle ear surgery is yet to be established. Further research is needed to make it clear if an endoscopic approach could possibly be superior to a microscopic approach in solving various technical difficulties in more complex procedures than myringoplasty, such as cholesteatoma surgery or congenital anomalies [6,7,31].

### Limitations of the Study

There are some limitations to our study that should be addressed in future studies. First, a lack of randomized controlled studies. The retrospective nature of all but one study included in our review is problematic in terms of controlling selection and allocation bias. For instance, in one study, it was suggested to the patients included in the EM group to use this specific approach in their cases in order to decrease postoperative pain [15]. Second, the risk factors that could influence surgical outcomes, such as age and size or site of the tympanic membrane perforation, were inconsistent among some of the included studies. Deviated outcomes are theoretically possible due to these uncontrolled factors. Finally, further larger cohort studies, ideally based on randomized controlled trials, are necessary to support our current interpretations.

## 5. Conclusions

The endoscopic approach allows the surgeon to perform a minimally invasive transcanal myringoplasty, avoiding surgical scarring, a temporary loss of cutaneous sensation and malposition of the ear, with comparable results to conventional MM in terms of the graft success rates and ABG gain, with a shorter operative time and fewer postoperative complications.

Since EM is an efficient minimally invasive technique, it should always be considered when performing myringoplasty in patients with COM.

## Figures and Tables

**Table 1:** Baseline characteristics.

Author	Year of Publication	Study Period	Type of Study	Total Patients (n)	EM Patients (n)	MM Patients (n)	EM Age (Years)	MM Age (Years)	EM Follow-Up (Months)	MM Follow-Up (Months)
Secaattin Gulsen, Adem Baltac [15]	2019	2015–2018	RNRC	126 (149 ears)	67 (78 ears)	59 (71 ears)	45.4 (15–61)	54.8 (18–72)	8.2	9.3
Ahmad Daneshi et al. [16]	2020	2014–2016	RNRC	130	75	55	39.85 (18–68)	38.25 (16–77)	12	12
Qimei Yanget et al. [17]	2022	2011–2016	RNRC	345	224	121	40.87 (18–67)	38.56 (18–66)	12.74 (6–48)	14.08 (6–36)
Chin-Kuo Chen et al. [18]	2022	2008–2018	RNRC	43	22	21	50.6	45.9	14	13.7
Tzu-Yen Huang et al. [19]	2018	2011–2014	RNRC	95 (100 ears)	47 (50 ears)	48 (50 ears)	54.2	49.9	6	6
A. C. Jyothi et al. [20]	2017	2011–2013	PRC	120	60	60	28.5	31.4	12	12

RNRC = Retrospective non-randomized comparative; PRC = Prospective randomized comparative; EM = Endoscopic myringoplasty; MM = Microscopic myringoplasty.

**Table 2:** Graft analysis.

Author	EM Graft Material	MM Graft Material	EM Graft Technique	MM Graft Technique
Secaattin Gulsen, Adem Baltacı [15]	Tragal chondroperichondrial	Tragal chondroperichondrial	Underlay	Underlay (postaural)
Ahmad Daneshi et al. [16]	0.5 mm thickness conchal chondroperichondrial	0.5 mm thickness conchal chondroperichondrial	Underlay	Underlay (postaural)
Qimei Yanget et al. [17]	Tragal chondroperichondrial	Temporalis fascia	Underlay	Underlay (postaural)
Chin-Kuo Chen et al. [18]	Postconchal perichondrium	Temporalis fascia	Over-underlay	Over–underlay (postaural)
Tzu-Yen Huang et al. [19]	Tragal perichondrium	Temporalis fascia	Underlay	Underlay (postaural)
A. C. Jyothi et al. [20]	Temporalis fascia	Temporalis fascia	Underlay	Underlay (postaural)

EM = Endoscopic myringoplasty; MM = Microscopic myringoplasty.

**Table 3:** Outcomes analysis.

Outcome	Graft Success Rate		Pre-op. ABG (dB)		Post-op. ABG (dB)		Mean Operative Time (min)	
Authors	EM	MM	EM	MM	EM	MM	EM	MM
Secaattin Gulsen, Adem Baltacı [15]	94.80%	92.90%	28.9	29.7	8.2	7.9	34.9	52.7
Ahmad Daneshi et al. [16]	97.30%	96.40%	25.2	24.9	15.7	14.4	76.7	161
Qimei Yanget et al. [17]	94.64%	90.90%	19.26	18.13	7.72	8.34	49.22	81.22
Chin-Kuo Chen et al. [18]	86.30%	85.70%	20.7	17.6	10.2	12.5	79.8	99.9
Tzu-Yen Huang et al. [19]	98%	98%	21.6	21.4	13.3	12.5	50.4	75.5
A. C. Jyothi et al. [20]	91.67%	93.30%	34.16	35.54	18	16	60	120

ABG = air–bone gap; EM = endoscopic myringoplasty; MM = microscopic myringoplasty.

## Data Availability

Data are available on request to the corresponding author.
